# Analysis of Bicycle-Motor Vehicle Crashes in San Antonio, Texas

**DOI:** 10.3390/ijerph18179220

**Published:** 2021-09-01

**Authors:** Khondoker Billah, Hatim O. Sharif, Samer Dessouky

**Affiliations:** Department of Civil and Environmental Engineering, University of Texas at San Antonio, San Antonio, TX 78249, USA; khondoker.billah@utsa.edu (K.B.); samer.dessouky@utsa.edu (S.D.)

**Keywords:** bicycle, motor vehicle, bicycle facility, logistic regression, bivariate analysis

## Abstract

Bicycling is inexpensive, environmentally friendly, and healthful; however, bicyclist safety is a rising concern. This study investigates bicycle crash-related key variables that might substantially differ in terms of the party at fault and bicycle facility presence. Employing 5 year (2014–2018) data from the Texas Crash Record and Information System database, the effect of these variables on bicyclist injury severity was assessed for San Antonio, Texas, using bivariate analysis and binary logistic regression. Severe injury risk based on the party at fault and bicycle facility presence varied significantly for different crash-related variables. The strongest predictors of severe bicycle injury include bicyclist age and ethnicity, lighting condition, road class, time of occurrence, and period of week. Driver inattention and disregard of stop sign/light were the primary contributing factors to bicycle-vehicle crashes. Crash density heatmap and hotspot analyses were used to identify high-risk locations. The downtown area experienced the highest crash density, while severity hotspots were located at intersections outside of the downtown area. This study recommends the introduction of more dedicated/protected bicycle lanes, separation of bicycle lanes from the roadway, mandatory helmet use ordinance, reduction in speed limit, prioritization of resources at high-risk locations, and implementation of bike-activated signal detection at signalized intersections.

## 1. Introduction

In recent years, bicycle ridership has gradually become one of the most common commuting means for the urban populace in the United States, as it is economical, energy-saving, and environmentally friendly. Many cities across the world are currently developing programs designed to promote bicycle riding as a means of reducing road congestions, controlling air pollution, and promoting healthier and more sustainable transportation alternatives [1,2]. Specific modifications of city bicycle infrastructure, including increasing bicycle lane mileage [3,4], adding bicycle share programs [5], and improving signage and street markings, are among the extensive integrated measures that proved to be most effective in increasing bicycle use [6]. However, bicyclists have a higher risk of severe crashes compared to vehicle drivers, which is a major deterrent to adopting bicycling as the main mode of transportation by many people [7,8]. For example, bicycle crashes were responsible for more than half million emergency visits to hospital in the United States in 2018 [9] and more than half million emergency department visits for traumatic brain injury during 2009–2018 [10]. According to the National Highway Traffic Safety Administration (NHTSA), the percentage of bicyclists in total fatalities has steadily increased from 1.8% to 2.2% between 2004 and 2013 [11]. Thus, the safety challenges associated with bicyclists remain a major concern in transportation planning. Several factors can contribute to bicycle injuries/fatalities and can be broadly classified as roadway-related, person-related, and environment-related. The common bicycle crash contributing factors include poor compliance with traffic laws and improper use of facilities, speeding, inadequate separation, crossing locations, inadequate conspicuity, and impairment and distraction [12].

San Antonio (located in Bexar County, Texas) is one of the fastest growing cities in United States and the seventh most populous with a population of 1.55 million [13]. In the year 2017, the population of San Antonio grew by 24,408 people, more than any other city in the United States [14]. The population of San Antonio is expected to increase by over one million by 2040 with a proportionate increase in the number of road users including pedestrians, bicyclists, and motorists. With more pedestrians, bicycles, and vehicles occupying the roads, conflicts on the road are also expected to increase. San Antonio is one of the premier places for bicycling in the United States, and the city has been enhancing its bicycle infrastructure by adding wider shoulders, multiuse paths, cycle tracks, bicycle lanes, bicycle boulevards, better routes, and shared lane markings, all of which are aimed at supporting bicycle traffic safety in the city [15]. In 2016, 62 bicyclists were killed in motor vehicle crashes in Texas, which was a 17% increase from 2015. San Antonio, being the second largest city in Texas, witnessed five deaths in 2016, a 25% increase from the previous year [16].

Researchers have used several techniques in traffic safety analysis models in previous studies including simultaneous equations [17], negative binomial [18,19,20], random effect ordered logit [21], ordinal probit [22], random effect negative binomial [23], and Bayesian hierarchical binomial and logistic models [24]. Logistic regression has also been widely used, especially in predicting motor-vehicle crashes by young drivers [25], determining the effect of age and/or gender on injury severity in head-on motor vehicle collisions [26], analyzing circumstances of bicycle crashes and injury pattern of cyclist casualties [27], improving motor vehicle/bicycle crash database [28], examining factors associated with bicycle injuries [29], determining risk factors related to e-bike and bicycle crashes [30], and determining the association of crash-related factors with mobile phone use for motorcyclists and e-bikers [31].

The safety of bicyclists can be enhanced by reducing collision risk and/or reducing the severity risk of a crash. Crash severity can be influenced by the behavior of the party at fault in a crash [32,33,34]. Pedestrian crashes support the hypothesis that the crash severity increases when the non-motorist is at fault, whereas bicycle crashes suggest otherwise [33]. Some studies also tried to determine the most suitable type of bicycle facility (e.g., on-street facility/shared-use path/curb lane) for safety and mobility [35,36], as well as the effect of specific roadway facilities [37,38]. However, studies focused on the effect of bicycle facilities (e.g., bicycle lanes, curb lanes, shared lane arrows, and road signage), as well as the effect of combination of these strategies, are still limited [6]. Previous studies found several factors to be significantly affecting bicyclist injury severity resulting from bicycle–motor vehicle crashes such as bicyclist race and gender, bicyclist old age, speeding, alcohol influence of driver/bicyclist, use of helmet, lighting condition, day of week, road type, and presence of intersection [12,33,39,40,41,42,43,44].

This study aims to contribute to a safer roadway for bicyclists by identifying high-risk locations and analyzing the effect of contributing factors on bicycle crash severity. This will assist the City of San Antonio, Texas Department of Transportation, and other traffic management stakeholders to prioritize allocation of available resources to high-risk locations, modify existing facilities at high-risk locations, adopt informed decisions regarding future designs, and run campaigns to the targeted audience. This study also contributes to the state of the literature by estimating the significant variables associated with different levels of injury severity of bicyclists, by examining how crash-related variables differ by party at fault for different injury levels, and how the presence of bicycle facility affects the severity of crash.

## 2. Materials and Methods

The crash data used in this study were acquired from the Texas Department of Transportation’s (TxDOT) Crash Records Information System (CRIS) for the 5 year study period (January 2014 to December 2018). Starting from 2003, all crashes that occurred on Texas roads and were reported by law enforcement officers, when predefined criteria were met, were included in the database. If any crash resulted in injury or death of any person involved or property damage ($1000 or more), a report was forwarded by the law enforcement officer to TxDOT no later than the 10th day after the date of the crash. The CRIS database includes the location and time of crash occurrence along with other relevant information pertaining to the crash (e.g., environmental, temporal, road, and bicyclists’ characteristics). The bike facility data were collected from the Traffic Engineering Division of the Transportation and Capital Improvements Department (TCI) of San Antonio. The facility demarcation is representative of the centerline of the facility.

Injury severity of the bicyclists was divided into two categories: KA (fatal or incapacitating injury; i.e., severe injury) and KAB (fatal or incapacitating or non-incapacitating injury; i.e., any confirmed injury). Bicycle crashes occurred on roads not maintained by the City of San Antonio were excluded from the analysis. Five datasets were prepared for analyses: all bicyclist-related crashes, bicyclist-not-at-fault crashes, bicyclist-at-fault crashes, on-facility bicycle crashes, and off-facility bicycle crashes. Any crash that occurred within 15 m of any side of the centerline of a road with a bicycle facility was considered as an on-facility crash. A bicyclist was assumed to be at fault if associated with any of the following primary contributing factors: changed lane when unsafe, disregarded stop and go signal, disregarded warning sign at construction, inattention, failed to control speed/speeding, disregarded stop sign or light, disregarded turn marks at intersection, faulty evasive action, fleeing or evading police, followed too closely, was drinking, overtook and passed with insufficient clearance, turned improperly, under influence of alcohol/drug, driving on wrong side/way, and was using a cellphone.

**Figure 1:**
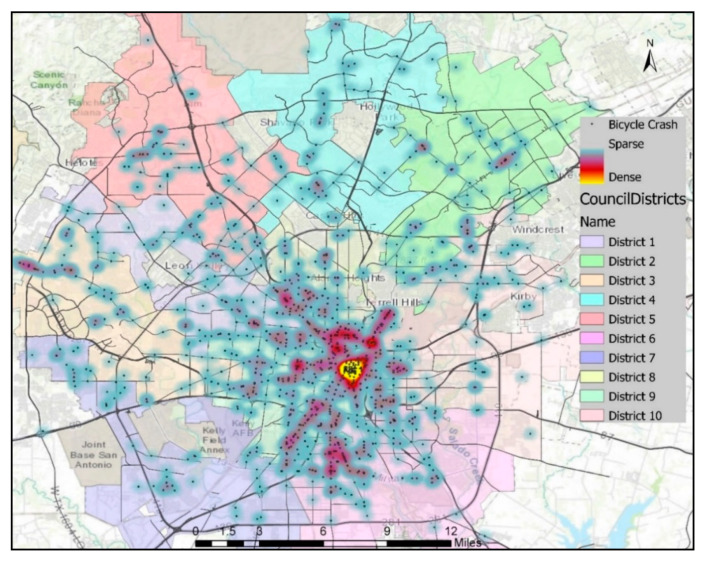
Heatmap of bicycle crashes in San Antonio based on crash density.

A heatmap (Figure 1) was created using reported locations of bicycle crashes to represent crash density over the study area. A color scheme consisting of a set of smoothly varying colors [45] was used for the density calculation with the kernel density estimation (KDE) method to visualize crash data as a continuous surface [46]. A defined kernel density surface is required for each crash point where the density value peaks at the center and gradually declines away from the center [47,48]. The KDE tool uses the quartic kernel function represented by the following equation:

(1) $$K_{2}\left(v\right)=\{\begin{array}{c} 3\pi^{-1}{\left(1-v^{T}v\right)}^{2}\;if\;v^{T}v<1\\ 0\;otherwise \end{array},$$

where $K_{2}\left(v\right)$ = is the kernel function for two-dimensional v. Generally, K is a radially symmetric unimodal probability density function [47].

The predicted density at a (p,q) location is determined by the following formula:

(2) $$Density=\frac{1}{{\left(radius\right)}^{2}}\overset{n}{\underset{i=1}{\sum }}\left[\frac{3}{\pi }\cdot pop_{i}{\left(1-{\left(\frac{distance_{i}}{radius}\right)}^{2}\right)}^{2}\right]For\;distance_{i}<radius,$$

where  i = 1,…,n are input points or point crashes $,\;pop_{i}$ is the population field value of point i, and $distance_{i}$ is the distance between point i and the location (p, q).

The hotspot analysis (Getis-Ord Gi*) was used to identify statistically significant spatial clusters [49]. This method uses the Getis-Ord Gi* statistic to identify clusters of high values (hotspots) and low values (cold spots). For each input feature, an output feature is created containing a *z*-score, *p*-value, and confidence level bin. The Getis-Ord local statistic is defined as

(3) $$G_{i}^{*}=\frac{\sum_{j=1}^{n}w_{i,j}x_{j}-\bar{X}\sum_{j=1}^{n}w_{i,j}}{S\sqrt{\frac{\left[n\sum_{j=1}^{n}w_{i,j}^{2}-{\left(\sum_{j=1}^{n}w_{i,j}\right)}^{2}\right]}{n-1}}},$$

where *x_j_* = the attribute value for feature *j*, *w_ij_* = the spatial weight between feature *i* and *j*, and *n* = the total number of features.

The average of the observed values is expressed as

(4) $$\bar{X}=\frac{\sum_{j=1}^{n}x_{j}}{n}.$$

The standard deviation is expressed as

(5) $$S=\sqrt{\frac{\sum_{j=1}^{n}x_{j}^{2}}{n}-{\left(\bar{X}\right)}^{2}}.$$

The complete spatial randomness (CSR) of the features or the values associated with those features is the underlying null hypothesis for this analysis. The null hypothesis is rejected when the *z*-score is relatively high/low and the *p*-value is very small, indicating statistically significant clustering or dispersion of features or values associated with features. The *p*-value represents the probability of randomness in clustering, and *z*-scores represent standard deviations. A very small *p*-value along with a very high/low *z*-score indicates the small probability of a cluster being a product of random distribution. When the search bandwidth is excessively large, a very smooth pattern is produced, making the process of differentiation harder between local hotspot locations. On the other hand, a spiky density pattern is produced by a narrow search bandwidth which highlights individual hotspot locations. Therefore, the use of an excessively large or narrow bandwidth might lead to false conclusions. To overcome this limitation, a trial-and-error method was adopted as recommended by previous studies [49,50,51]. When working with point data, a fixed distance band is suitable in the conceptualization of spatial relationships, and the selected threshold distance was 250 m.

The heatmaps derived using crash density cannot identify severe crash-prone locations. Hot/cold spots can be identified on the basis of crash severity, and each crash must be provided a weight based on its severity. The “compromise approach” (more severe crashes are provided with greater weight) has been popular in providing weight to crashes, but there is no established standard weighting system [52]. Crashes involving a fatality, serious injury, other injury, and property damage only were assigned severity indices of 3.0, 1.8, 1.3, and 1.0, respectively, by the Roads and Traffic Authority of New South Wales [53]. Another study based on Flanders, Belgium, used 5.0, 3.0, and 1.0 as severity indices for fatal, serious, and light injury crashes, respectively [54]. This study was primarily focused on identifying the high-risk locations for bicyclists in its spatial analysis segment and placed relatively greater weights on severe bicycle crashes. The following equation was used to determine the severity index (SI) of any location:

(6) SI=5.0×Y1+3×Y2+1.8×Y3+1.3×Y4+Y5,

where *Y*1, *Y*2, *Y*3, *Y*4, and *Y*5 represent the total number of crashes involving a fatality, serious injury, non-serious injury, possible injury, and no injury, respectively.

Bicycle–motor vehicle crash-associated variables selected for statistical analyses (Table 1) were based on a literature review [12,30,33,39,40,42,43,44,55] to study their effect on the severity of bicycle crashes as stand-alone variables and in conjunction with other. Bivariate analysis is utilized as an exploratory tool for hypothesis of the association test between a dependent and an independent variable, and it was used in this study to explore the relationships between the bicyclist injury severity and bicycle–motor vehicle crash-associated variables (as standalone variables). Chi-squared tests were performed for each categorical variable in the bivariate analysis to determine statistically significant differences within two or more classes in the distribution of the variable. Although a chi-squared test can determine the association between two variables, it fails to account for possible confounding factors. Hence, a definite causal relationship between two variables cannot be established from the chi-squared test. The strength of association was determined using the odds ratio (OR), which represents the ratio of the odds of an event occurring in the presence of the independent variable compared to the odds of that event occurring in the absence of that independent variable. Crash severity was used as the response variable during the development of logistic regression models to test the relationship of bicycle crash severity with other crash-associated variables (weather condition, lighting condition, speed limit, road class, collision type, time, period of week, month, and intersection presence) and bicyclist-related variables (age, gender, ethnicity, and helmet wearing practice) for all five data types described above. Developed logit models were used to identify statistically significant classes within the selected variables (in terms of crash severity) and to check the strength of association of each significant class in the logit model. This study used the logit as the natural logarithm of the odds, as shown in Equation (7).

Logit (A) = In (A/1 − A) = β_0_ + β_1_ × Z_1_ +…. + β_i_ × Z_i_, (7)

where A is the probability of severe crashes, Zi is the independent variable, and βi is the model coefficient directly determining the odds ratio.

The injury severity is determined by the officer on duty without cross-checking with hospital data or other external sources, and the party at fault is determined on the basis of the contributing factors reported by the officer; both can be subject to errors. Motor vehicle–bicycle crashes resulting in minor injuries or no visible injuries were often underreported, which might have led to a bias in results. Traffic volume data were not included in the spatial analysis, and only basic demographic variables were included due to the unavailability of city-wide detailed traffic volume data. The traffic policies and environmental factors did not change significantly during the study period to our best knowledge. However, consideration of the dataset as static over the study period might have been a potential limitation of this study due to the lack of control of environmental factors. Bias in sample selection was also possible, which is often associated with crash data analyses as people involved in crashes might not be representative of the general road user population. Coordinates were unavailable for 17.6% of the crashes; thus, the heatmap and hotspots were prepared using 83.4% of all crashes only.

## 3. Results and Discussion

### 3.1. Spatial Analysis

**Figure 2:**
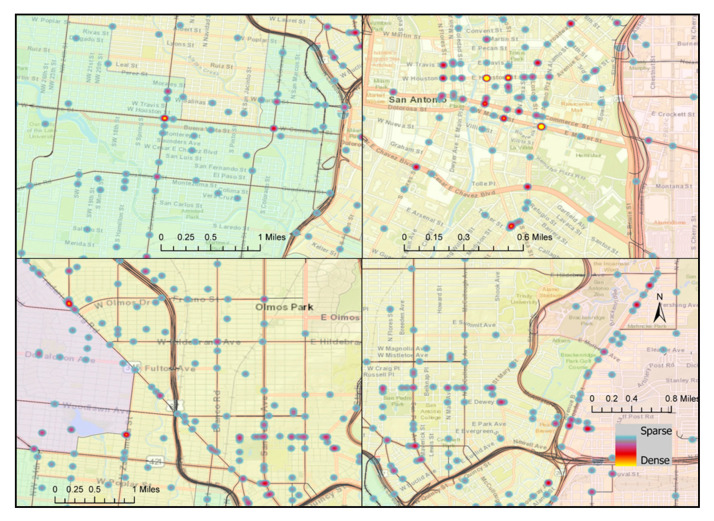
Locations with frequent bicycle crashes: intersection at W Commerce St and Zarzamora St (**top left**), intersections in the city center (**top right**), intersection at Fredericksburg Rd and San Pedro Ave (**bottom left**), and intersection at Broadway St and W Ashby Pl (**bottom right**).

**Figure 3:**
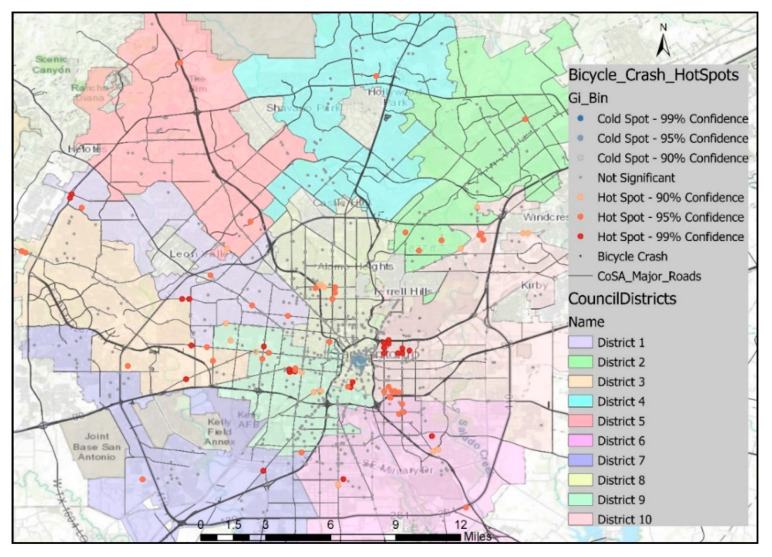
Hot and cold spots of bicycle crashes in San Antonio based on crash severity.

**Figure 4:**
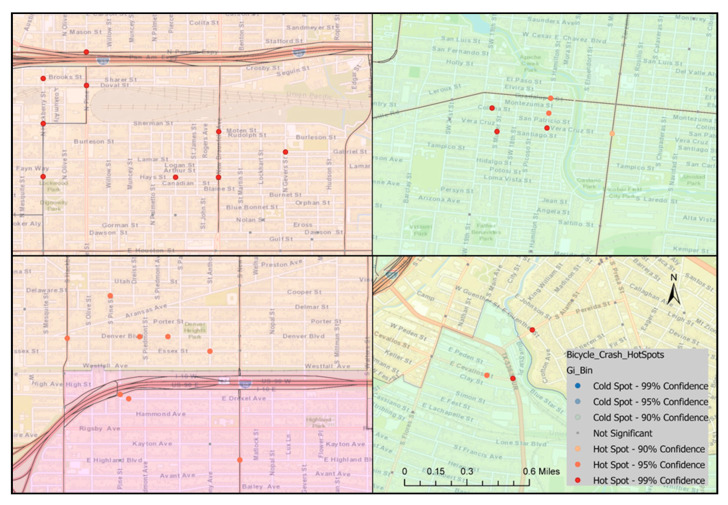
Locations with statistically significant cluster of bicycle crashes near the intersections at N New Braunfels Ave and Hays St (**top left**), Guadalupe St and S Hamilton St (**top right**), S Hackberry St and Denver Blvd (**bottom left**), and E Cevallos St and TX-536 SPUR (**bottom right**).

Crashes were analyzed by location and severity to identify areas with relatively higher densities of severe crash occurrences, as shown in Figure 1, Figure 2, Figure 3 and Figure 4. The downtown area had the highest bicycle crash density. This was expected since the downtown area experiences the highest bicycling activities. The intersection at E Houston St and N St Mary’s St was one of the most critical in the City in terms of crash frequency, experiencing 10 bicycle crashes during the study period. Another location of high crash frequency was the intersection at E Market St and N Alamo St.

The spatial distribution of locations with high bicycle crash density in the heatmap and locations with a statistically significant cluster of severe bicycle crashes with complete spatial randomness in hotspot analysis did not superimpose (Figure 1, Figure 2, Figure 3 and Figure 4). However, cold spots in terms of crash severity were centered at some of the intersections at E Houston St, E Commerce St, and N St Mary’s St in the heart of the city, which is the area with the highest bicycle crash densities (Figure 3). Traffic speed is relatively slow in the downtown area, and bicycle facilities were more prevalent. These might be the factors resulting in statistically less severe crash locations in this area. On the other hand, hotspots were observed at locations with comparatively lower crash frequencies and higher road speed limits.

### 3.2. General Characteristics of Bicycle Crashes

Bicycle crashes accounted for a total of 1528 crashes (1539 bicyclists involved) in San Antonio during the study period, accounting for 0.84% of the total crashes. However, bicycle crashes accounted for 3.9% of all fatal and incapacitating injury crashes. These crashes resulted in death of 16 bicyclists and another 105 bicyclists sustained incapacitating injuries. Bicyclists were at fault in 569 crashes (37.2%), and these crashes resulted in slightly fewer KA injuries compared to crashes where bicyclists were not at fault (6.7% vs. 8.6%). Crash coordinates were available for 1251 crashes (296 on-facility crashes and 955 off-facility crashes). The introduction of a bicycle facility had no statistically significant effect on the injury severity of bicyclists.

**Figure 5:**
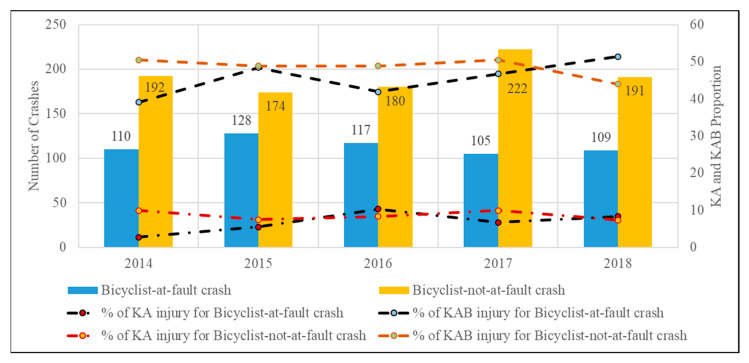
Annual frequency and proportions of KA and KAB injury of bicycle crashes based on party at fault.

**Figure 6:**
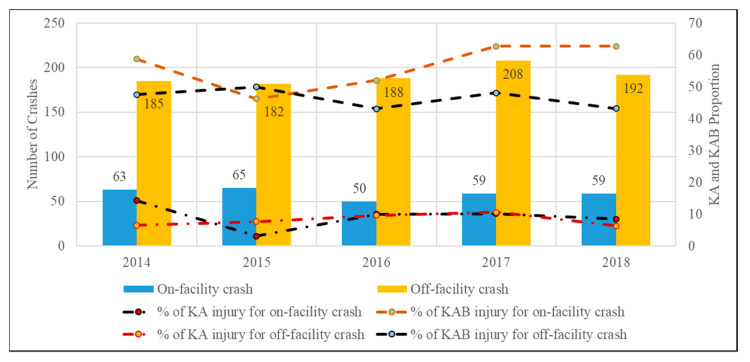
Annual frequencies and proportions of KA and KAB bicycle crashes based on presence of facility.

Figure 5 and Figure 6 show the frequencies of bicycle crashes and the KA and KAB injury proportions over the years based on the party at fault and the presence of facility, respectively. The proportion of KA and KAB injuries slightly increased over time for bicyclist-at-fault crashes. The proportion of on-facility KA and KAB injury crashes dropped in 2015 but experienced an overall increase in the subsequent years (Figure 6). The increase in the total number of bicycle crashes in 2017 could be attributed to the introduction of more bike lanes, thus attracting more bicyclist ridership [56].

**Figure 7:**
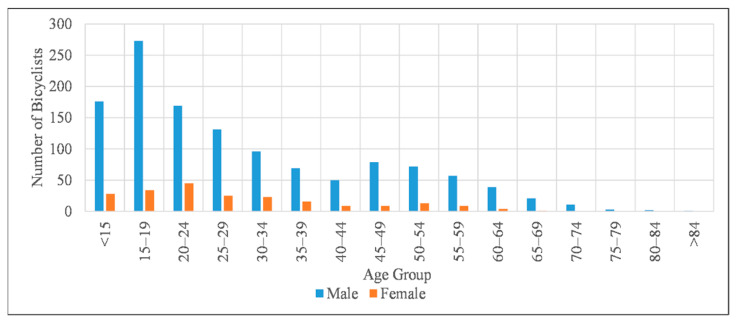
Age and gender distribution of bicyclists involved in crashes.

The age of 73 bicyclists was not specified in CRIS. Among the 1466 bicyclists with known age, 1249 were males, 216 were females, and the gender of one bicyclist was not reported. Crash frequency with respect to age and gender for each age group is shown in Figure 7. Male bicyclists were more common victims for all age groups, especially for older groups. The 20–24 age group had a noticeably higher proportion of female bicyclists involved in crashes. The frequencies and proportions of bicycle crashes based on two different crash severity levels for potential crash contributing factors and the statistically significant differences between classes of selected variables are presented in Table 2 and Table 3 (proportions expressed in boldface type indicate a statistically significant difference, i.e., *p* < 0.05, whereas italicized type indicates a marginally statistical significance, i.e., 0.05 < *p* < 0.1).

### 3.3. Bivariate Analysis

#### 3.3.1. Environmental Factors

As expected, a substantial proportion of bicycle crashes (69.4%) occurred in daylight conditions (Table 2). Except for on-facility crashes, all other crash types were associated with higher proportions of KA injury in dark lighting conditions (“dark, lighted” and “dark, not lighted”). Dark lighting conditions significantly increased KA injury risk for all bicyclist, bicyclist-not-at-fault, and off-facility crashes. The odds ratio of severe bicycle injuries was over 1.5 times in dark lighting conditions for all crash types except for on-facility crashes (OR 0.9).

For both injury severity levels (KA and KAB), the crash severity risk in dark lighting conditions was higher for bicyclist-not-at-fault crashes compared to bicyclist-at-fault crashes (OR 2.2 vs. OR 1.5 for KA crashes and OR 1.3 vs. OR 1.1 for KAB crashes). For KAB injury, the effect of lighting condition was statistically significant only when bicyclists were not the party at fault. Bicyclists being unaware of an imminent crash, collision type/angle, and faulty behaviors by motor vehicle drivers such as distracted driving, drugs and alcohol, and speeding might have contributed to the greater risk associated with crashes where bicyclists were not at fault in dark conditions. Dark lighting conditions increased the risk of KA injury more than non-incapacitating injury. The greater reaction time required by both motor vehicle drivers and bicyclists to avoid collision under reduced visibility might make the collision more lethal and, hence, increase injury severity.

Only 3.2% of the crashes occurred in adverse weather (rain/hail/snow/fog/crosswind). Adverse weather conditions increased the overall KA injury risk. Bicyclist-not-at-fault crashes had relatively greater KA injury risk. None of the only five crashes that occurred on roads with bicycle facilities during adverse weather resulted in KA injury. Adverse weather conditions increased the odds of KA injury (all crashes) but decreased the odds of KAB injury (bicyclist-at-fault crash being the exception).

#### 3.3.2. Time Factors

The weekend period, especially Sunday, had high proportions of KA injury, but relatively lower crash frequency (Table 2). For KAB injury, however, only crashes where bicyclists were not at fault had higher injury risk during the weekend. Bicyclists showed lower faulty behavior for weekend crashes (31.4% during weekend vs. 39.0% during weekdays). The day of crash occurrence had a statistically significant effect on KA injury for all bicyclist, bicyclist-not-at-fault, and off-facility crashes. All five crash types had increased KA injury risk during the weekend. Lower traffic volumes on roads leading to the higher speed of motor vehicles during the weekend might have contributed to the higher KA injury risk. KA injury OR was higher for bicyclist-not-at-fault crashes compared to bicyclist-at-fault crashes (1.9 vs. 1.3) during the weekend. The results suggest that the injury severity of bicyclists during weekend was more influenced by the fault of motor vehicle drivers rather than the fault of bicyclists. The association of the day of the week with KAB injury was not statistically significant for any of the five types of crashes.

About 21% of all bicyclist crashes occurred during the nighttime (8 p.m.–6 a.m.), as shown in Table 2. Nighttime was associated with higher proportions of KA and KAB injury for almost all crash types (KAB injury for on-facility crashes being an exception). Off-facility and bicyclist-not-at-fault crashes had higher proportions of KA injury during the nighttime compared to their counterparts. KA injury was significantly influenced by the time of crash occurrence. The risk of KA injury increased during the nighttime for all crash types. On-facility crashes had the lowest association with KA risk and the lowest KA proportion during nighttime among all five crash types (lowest nighttime OR, 1.5). The majority of on-facility crashes occurred at intersections, and bicycle crashes occurring at intersections had a substantially low risk of severe injury. Moreover, on-facility roads have dedicated facilities for bicyclists such as separated bike lanes and markings, and they are often amply illuminated, which might have contributed to the reduced severe injury risk of bicyclists on these roads. The KAB proportion also increased during nighttime (except for on-facility crashes), but to a relatively smaller magnitude than the KA proportion. Off-facility crashes had a higher injury risk during nighttime irrespective of injury severity. The presence of bicycle facilities on roads might have decreased the severity of nighttime crashes for these roads.

Bicycle crashes were more frequent during the spring and less frequent during the winter season (Table 2). Summer had relatively higher KA (except for bicyclist-at-fault crashes) and KAB (except for on-facility crashes) proportions. The relationship between the season and crash occurrence and severity was statistically significant only for all and bicyclist-not-at-fault crashes (KAB proportion). The risk of KA injury on on-facility roads, however, substantially decreased during winter (3.4% in winter vs. 12.3% in summer; OR 0.3 in winter vs. OR 1.2 in summer). Lower temperatures in the winter might encourage more bicyclists to wear helmets, and lower cycling practice during winter might make sharing of the roadway easier, which might have contributed to the reduced KA injury. KAB injury risk significantly increased during the June–November period for crashes where bicyclists were not at fault, which might be attributed to the lighter clothing practice and less use of protective gear by bicyclists.

#### 3.3.3. Road Factors

Among all bicycle crashes, 78.6% occurred on roadways with a speed limit ≥25 mph and 13.2% occurred on roadways with a speed limit <25 mph (Table 2). As expected, crashes on roads with a higher speed limit resulted in higher KA and KAB proportions, which is consistent with previous findings [42,43]. The association of speed limit with KA/KAB injury was not statistically significant for any of the five crash types. However, all bicyclist and bicyclist-not-at-fault crashes had a significant association with KAB injury. In general, the higher speed limit increased the risk of KA injury more than KAB injury as observed from OR values. When bicyclists were not at fault on roads with a higher speed limit, crashes tended to result in more severe injuries for both levels of injury severity, implying that the fault of motor vehicle drivers had a greater influence on injury severity. For roads with a higher speed limit, the odds of KA injury were greater for on-facility crashes compared to off-facility crashes (OR 1.7 vs. OR 0.8). Minor roads, nontraffic ways, and the majority of the city roads do not have bicycle facilities in general, and these roads usually have lower speed limits, leading to the reduced KA crash risk for off-facility roads.

About half (49.3%) of all crashes involving bicyclists occurred at intersections (Table 2), whereas intersection crashes represented only 31.2% of all crashes in San Antonio. Vehicles coming from multiple directions and higher traffic volumes at intersections might have contributed to bicycle crashes at intersections. For all five types of crashes, the presence of an intersection decreased the severity of the crash (lower KA proportion). However, only bicyclist-not-at-fault and on-facility crashes had lower KAB proportions. Collisions during turning were found to be less lethal than collisions while driving straight, and a large proportion of the bicycle crashes at intersections occurred while taking a left or right turn. Less lethal collisions while turning, along with the reduced speed of vehicles at intersections, might contribute to lower KA injury risk at intersections. The risk of KA injury was slightly greater at intersections when bicyclists were not at fault (OR 0.8 for bicyclist not at fault vs. OR 0.6 for bicyclist at fault). Intersection presence substantially reduced KA injury, but slightly increased non-incapacitating injury severity. Apart from driver inattention, the most common fault of motor vehicle drivers was failure to yield right of way, whereas, for bicyclists, it was disregard of stop signal/sign and failure to yield. When a bicyclist at fault is driving straight at a stop sign/signal, the motor vehicle is just beginning to gain speed in most cases. On the other hand, when a bicyclist is taking a turn and the motor vehicle is moving at a greater speed, the collision between motor vehicle and bicycle rarely occurs at a right angle. These might have been the reasons behind the relatively reduced injury risk for bicyclist-at-fault crashes at intersections.

About two-thirds of all bicycle crashes (67.7%) occurred when the bicyclist was going straight (Table 2), whereas 29.9% crashes occurred while taking a turn (13.7% right turn, 16.2% left turn). For all five bicycle crash types, KA injury was highest when the bicyclist was going straight, followed by when turning left (bicyclist-at-fault crashes being an exception). The association of collision type with KA/KAB severity was not statistically significant for any of the crash types. In cases of crashes where bicyclists were not at fault, collision during a turn had a greater non-incapacitating injury risk. When the bicyclist was driving straight, the motor vehicle involved in the crash was going parallel to the bicycle (hitting the bicycle from the side/back), approaching from a perpendicular direction at crossings, or in some cases coming from opposite direction. In cases of crashes when the vehicle was moving parallel to the direction of bicycle, the speeds of the vehicle and bicycle were greater than that when turning, which might have resulted in more severe crashes. For perpendicular and opposite direction crashes, the severity was expected to be even higher.

Although the majority of crashes occurred on city streets (67.8%), crashes occurring on highways (9.4%) had higher KA proportions in general (Table 2). When bicyclists were at fault in crashes on highways, the proportion of KA injury was substantially lower (13.3% overall vs. 4% for bicyclist-at-fault crashes). KA injury risk increased significantly on highways for crashes where bicyclists were not at fault (OR = 2.1). Bicyclist-at-fault crashes had lower fatal/incapacitating injury and higher non-incapacitating injury proportions on highways and farm-to-market (FM) roads compared to other road types, implying that the fault of motor vehicle drivers in a crash increased the risk of injury severity on roads with a higher speed limit. Higher vehicle speeds on highways and faulty motor vehicle drivers unable to properly share the roadway with bicycles off facility increased the injury risk the most.

#### 3.3.4. Bicyclist Factors

Consistent with a previous study, the absence of a helmet increased the odds of severe injury of bicyclists [57]. Use of a helmet was scarce among bicyclists involved in crashes (14.6%), as shown in Table 3. Except for the KA proportion for off-facility crashes (9.0% with helmet worn, 8.6% not worn), the absence of helmet use was associated with higher severe injury proportions for all five crash types. In the absence of helmet, KA injury proportions substantially increased for bicyclist-at-fault crashes (7.6% vs. 2.0%) and on-facility crashes (10.5% vs. 6.6%). For all five crash types, bicyclists helmet status had a greater association with non-severe injury crashes compared to severe injury crashes. When bicyclists were at fault in a crash and did not wear a helmet, the odds of KA injury were almost four times greater compared to crashes where bicyclists were not the party at fault, implying that the combination of bicyclist’s fault and absence of a helmet increases crash severity.

Mixed results were observed regarding the association of bicyclist ethnicity with bicyclist injury severity in previous studies. One study using bicyclist crash data of Los Angeles concluded that Hispanic bicyclists were less likely to sustain severe injuries [12] while another study based on bicyclists of Colorado suggested that Hispanic bicyclists had a higher fatality rate [58]. More than half of the bicycle crash victims in San Antonio were Hispanic (53.7%), followed by Whites (34.8%) and Blacks (6.5%). This ethnic distribution does not exactly mirror the community distribution (54% Hispanics, 24.7% Whites, and 6.5% Blacks) implying that Whites are overrepresented in bicycle crashes and maybe bicycle use. The proportions of victims from Asian and other ethnicities were extremely small. For all crash types, Hispanics were associated with substantially lower severe injury and relatively lower non-severe injury. When bicyclists were at fault, Black bicyclists were relatively more susceptible to severe injury. When bicyclists were not the party at fault and crash did not occur on roads with bicycle facility, Hispanics had significantly lower severe injury risk. When bicyclists were at fault, the odds ratio of severe injury for non-Hispanics (White, Black, Asian, or other) was 1.0, implying the neutrality of ethnicity in affecting crash severity when bicyclists were at fault in crashes. A higher proportion of young Hispanic bicyclists, a relatively lower proportion of Hispanic bicyclists (especially older Hispanic bicyclists) on highways and FM roads (especially during weekend), and relatively more helmet use during nighttime were some of the reasons behind the lower KA crash risk for Hispanics.

The vast majority of bicyclists involved in crashes were male (84.9%). When bicyclists were at fault and crashes occurred on roads equipped with bicycle facilities, male bicyclists were more likely to be involved in severe crashes and female bicyclists were more likely to be involved in non-severe crashes, which is consistent with a previous study [12]. Overall, the effect of the bicyclist’s gender was more profound for non-incapacitating injury severity (although not statistically significant). Older bicyclists (age ≥65) were more susceptible to KA injury than younger bicyclists (12.8% vs. 4.5%). All severe injury crashes related to older bicyclists occurred on roads without bicycle facilities, and advanced age of the bicyclist was a strong predictor of severe bicyclist injury, consistent with previous studies [33,42]. The greater reaction time required by older drivers along with being more vulnerable physically might have been the reason behind the higher KA injury risk. On-facility crashes resulted in greater non-severe injury proportions for all age groups, which might reflect the effectiveness of facility implementation in reducing severe injury crashes. The age of bicyclists had a greater association with KA injury compared to KAB injury. The odds of KA injury were significantly lower for young drivers when motor vehicle drivers were at fault, possibly due to better physicality and adaptivity of young bicyclists in crash situations and cycling on relatively safer roadways.

### 3.4. Logistic Regression Results

Logistic regression models were developed using bicyclist, environment, road, and temporal variables associated with bicycle–motor vehicle crashes to analyze their effect on severity of bicyclist injury. Typically, bivariate analysis identifies key predictors discretely, while logistic regression identifies key predictors taking all variables into consideration. A pair of logit models were constructed for each combination of datasets and injury severity level, one with bicyclist-related variables and the other one with the remaining variables. The coefficient estimates in log odds terms with the respective reference category, significance, standard error, and odds ratio are presented in Table 4, Table 5 and Table 6. The signs and values of coefficient estimates and the difference among the coefficient estimates of the categorical variables are indicative of the effects of the categorical variables on the crash severity. A negative estimate coefficient indicates a decrease in the odds of severe injury of a bicyclist, while a positive estimate coefficient suggests an increase in the odds of a severe injury. For example, when the KA injury of all bicyclists was considered (Table 4), daylight conditions experienced a negative coefficient (−0.51) with dark lighting conditions as the reference, implying that a change from dark lighting conditions to daylight conditions decreased the log odds of a severe bicyclist injury by 0.51. The asterisk sign associated with the coefficient estimate (*) indicates that daylight conditions significantly (*p* < 0.05) reduced severe bicyclist injury. Similarly, the positive coefficient (0.52) and significance (**) associated with the non-Hispanic ethnicity implies that non-Hispanic bicyclists had significantly (*p* < 0.01) higher odds of sustaining severe injury.

The results of logistic regression were consistent with the bivariate analysis in general. The statistical significance of some variables was different between bivariate analysis and logistic regression, but the relationships between bicycle crash severity and different variables exhibited a similar direction regardless of crash severity level, party at fault in a crash, and bicycle facility status. Bicyclist age and ethnicity, day of week, month, intersection presence, and lighting condition significantly influenced the KA injury of bicyclists, whereas bicyclist gender, helmet use, and collision type significantly influenced the KAB injury of bicyclists. Older bicyclists (age ≥ 65) were more susceptible to KA injury (OR 3.2) compared to young bicyclists (age ≤ 18). The risk of sustaining a severe injury was almost twofold for non-Hispanic bicyclists compared to Hispanic bicyclists. Further analyses revealed that the high proportion of young bicyclists among crashes involved Hispanic bicyclists, relatively greater use of a helmet by Hispanic bicyclists during nighttime, and fewer crash incidents on highways and FM roads involving Hispanic bicyclists were the reasons behind the relatively lower KA injury severity risk for Hispanic bicyclists. The weekend period significantly increased the KA injury risk of bicyclists (OR 1.6), possibly due to the high proportion of DWI incidents and greater vehicle speeds due to lower traffic volumes. Intersection presence and daylight conditions reduced the KA injury risk of bicyclists. Although wearing a helmet did not significantly reduce the KA injury risk, the KAB injury risk was significantly reduced (OR 0.7). This demonstrates effectiveness of helmet use in reducing, at least, minor injuries.

Except for the bicyclist age, none of the factors significantly influenced the KA injury resulting from on-facility crashes, implying the effectiveness of bicycle facilities in minimizing the influence of these factors on KA injury. A higher speed limit (>25 mph) on roads substantially increased the odds of KA injury and KAB injury in the absence of bicycle facilities. Compared to the likelihood of KAB injury during daytime (OR 1.0), nighttime (6 p.m.–6 a.m.) KAB injury risk was higher (OR 1.4) for on-facility crashes and lower (OR 0.7) for off-facility crashes. Similarly, the risk of KA injury was more than doubled during nighttime for bicycle crashes occurring on roads with bicycle facilities compared to off-facility roads. Unlike roads with bicycle facilities, other roads with a higher speed limit significantly increased the KAB injury risk, which should encourage greater implementation of bicycle facilities. Non-Hispanics experienced significantly high severe injury risk on on-facility roads and non-severe injury risk on off-facility roads.

## 4. Conclusions

This study examined the spatial distribution of bicycle crashes and analyzed the factors that influence the injury severity of bicyclists involved in bicycle–motor vehicle crashes using 5 year crash data for San Antonio, TX. Bivariate analysis and logistic regression modeling were used to examine the relationships among different human-, environment-, and crash-related factors for two injury severity levels. Bicyclist age and ethnicity, lighting condition, road class, time of day, and day of the week had the most significant association with severe bicycle crashes. Overall, bicycle crashes resulting in severe injury of bicyclists were more strongly influenced by the variables studied compared to non-severe crashes.

In the absence of bicycle facilities, severe bicycle crashes had several strong predictors (bicyclist age and ethnicity, intersection presence, and temporal variables), while on-facility severe bicycle crashes had almost none, implying the effectiveness of bicycle facilities in reducing the influence of some variables on crash severity. Facilities such as separate/protected bicycle lane might contribute to decreasing the chance of a deadly collision with motor vehicles, thereby reducing severe injury risk. Although the presence of bicycle facilities reduced the effects of some variables to some extent, their presence made no statistically significant difference in crash severity when compared to roads without bicycle facilities.

Wearing a helmet is not mandatory for bicyclists in Texas [59], but some major cities in Texas have ordinances of mandatory helmet use. The results indicate very limited practice of helmet use among crash involved bicyclists in San Antonio and an increase in the injury severity of bicyclists in the absence of a helmet for bicyclist-at-fault and on-facility crashes. Our findings suggest that older, male, and non-Hispanic bicyclists of San Antonio are more likely to sustain severe injuries. Identifying zones with a higher proportion of older bicyclists and introducing bicycle facilities in these zones, a targeted campaign to encourage the wearing of helmets and protective gear, and the introduction of a mandatory helmet use ordinance in San Antonio might reduce the severe injuries of older bicyclists.

Bicycle crashes on roads with a relatively higher speed limit (e.g., highways and FM roads) notably increased the bicyclist injury severity risk, especially for crashes where bicyclists were not at fault and those which occurred on roads with bicycle facilities. Identification of the optimal bicycle lane width and separation of bicycle lanes from the roadway were effective in reducing the crash rate [35] and raised bicycle crossings were effective in increasing the safety of bicyclists [60]. These techniques should be introduced to roads with a relatively higher speed limit and bicycling activities.

The weekend period had a relatively lower bicycle crash count but higher severe injury proportion. Nighttime during weekend period had a substantially high severe injury risk, probably due to the higher frequency of DWI and distracted drivers [33], encouraging the use of ridesharing services during this period. Bicyclists exhibited reduced faulty behavior during the weekend, whereas the proportion of faulty motor vehicle drivers of San Antonio was greater during the weekend [44].

The relatively higher bicyclist injury risk during summer and lower injury risk during winter might be attributed to the seasonal differences in clothing practice. Bicycle crashes, especially on-facility bicycle crashes, occurred at a much higher proportion at intersections. This might be an indicator of the effectiveness of facility implementation, as fewer crashes occurred on roadway segments where facilities were introduced.

Intersections in the city center were more prone to a higher bicycle crash frequency but lower injury severity of bicyclists, which is analogous to the overall crash pattern at intersections in San Antonio [41]. The primary contributing factors to bicycle–motor vehicle crashes were driver inattention and disregard of stop sign/light for both bicyclists and vehicle drivers. Implementation of bike-activated signal detection and bicycle signal heads at signalized intersections, encouraging and endorsing the use of autonomous vehicles, as well as the implementation of an automated red-light camera and shared marking lanes along with campaigns toward targeted audience, can significantly reduce bicycle crash incidents. Future studies focusing on the in-depth analysis of the most significant variables might be helpful in providing case-specific recommendations.

## Figures and Tables

**Table 1:** Description of the logistic regression independent variables.

Num.	Description	Values	Num.	Description	Values
1	Day of Week	Weekend	7	Speed limit	≤25 mph
		Weekday			>25 mph
2	Season	Winter	8	Collision type	Going straight
		Spring			Turning
		Summer			Other roads
		Fall	10	Gender	Male
3	Time of Day	8 p.m.–6 a.m.			Female
		6 a.m.–8 p.m.	11	Age	≤18
4	Lighting condition	Daylight			19–64
		Dark			≥65
5	Weather condition	Rain	12	Ethnicity	Non-Hispanic
		No rain	13	Helmet status	Wearing
6	Intersection presence	Yes			Not wearing
		No			

**Table 2:** Proportions of bicyclist KA and KAB crashes in San Antonio from 2014–2018 corresponding to environmental, temporal, and road-related variables—Inter/Y and Inter/N denote at and not at intersection, respectively; 25 mph refers to the roadway speed limit.

	All Bicyclists			Not at Fault			At Fault			On Facility			Off Facility		
	*N*	KA	KAB	*N*	KA	KAB	*N*	KA	KAB	*N*	KA	KAB	*N*	KA	KAB
Overall	1528	7.9	47.4	959	8.7	48.6	569	6.7	45.5	296	9.1	56.4	955	8.4	46.6
Daylight	1061	**6.4**	*45.4*	640	**6.6**	*45.6*	421	6.2	45.1	199	9.0	55.3	644	**6.5**	44.9
Dark	417	**12.0**	*50.6*	286	**13.3**	*52.1*	131	9.1	47.3	87	8.0	57.5	282	**13.1**	48.9
Rain	49	12.2	40.8	38	13.2	36.8	11	9.1	54.5	5	NA	40.0	35	8.6	40.0
No rain	1473	7.8	47.7	915	8.6	49.1	558	6.6	45.3	290	9.3	56.6	916	8.4	46.9
Highway/FM	*202*	*10.4*	*52.5*	143	**12.6**	54.5	59	5.1	47.5	20	10.2	54.8	180	10.5	*52.2*
Other roads	*1299*	*7.3*	*46.6*	700	**8.1**	48.2	499	7.0	36.8	273	8.8	54.5	747	7.6	*45.0*
≤25 mph	202	5.4	*40.6*	210	8.1	*46.2*	70	5.7	41.4	17	5.9	58.8	71	9.9	38.0
>25 mph	1202	8.2	*48.3*	749	8.8	*49.3*	453	7.3	46.6	264	9.5	56.8	816	8.3	47.2
Weekend	353	**11.3**	49.5	242	**12.8**	52.9	111	8.1	42.4	70	14.3	57.2	217	**12.0**	49.3
Weekday	1175	**6.9**	46.8	717	**7.3**	47.2	458	6.3	46.3	226	7.5	56.2	738	**7.3**	45.8
Inter/Y	754	6.8	47.7	523	7.8	47.5	318	5.3	48.1	208	7.7	54.3	513	**6.6**	46.6
Inter/N	774	9.0	47.2	436	9.4	49.5	251	8.4	42.2	88	12.5	61.4	442	**10.4**	46.6
Winter	319	6.9	**43.3**	213	7.5	**45.1**	106	5.7	39.6	58	3.4	50.0	203	8.4	44.8
Spring	421	6.9	**45.1**	252	6.3	**43.4**	169	7.7	47.9	81	8.6	58.0	270	6.3	43.3
Summer	384	9.4	**52.6**	233	10.7	**54.9**	151	7.3	49.0	81	12.3	56.8	242	9.5	51.2
Fall	404	8.4	**48.3**	261	10.0	**51.0**	143	5.6	43.4	76	10.5	59.2	240	9.6	47.1
8 p.m.–6 a.m.	322	**13.0**	*52.5*	219	**13.7**	*7.2*	103	**11.7**	49.5	76	11.8	52.6	212	**14.2**	51.4
6 a.m.–8 p.m.	1206	**6.6**	*46.1*	740	**7.2**	*47.0*	466	**5.6**	44.6	220	8.2	57.7	743	**6.7**	45.2
Straight	1034	8.8	47.8	604	9.9	48.0	430	7.2	47.4	183	10.4	60.1	637	9.6	46.8
Turning	457	6.6	48.1	330	7.0	50.9	127	5.5	40.9	113	7.1	50.4	300	6.4	47.6

**Table 3:** Proportions of bicyclist KA and KAB crashes in San Antonio from 2014–2018 corresponding to bicyclist-related variables.

	All Bicyclists			Not at Fault			At Fault			On Facility			Off Facility		
	*N*	KA	KAB	*N*	KA	KAB	*N*	KA	KAB	*N*	KA	KAB	*N*	KA	KAB
Overall	1539	7.9	47.6	966	8.6	48.7	573	6.6	45.6	296	9.1	56.4	963	8.4	46.6
Helmet/Yes	224	7.1	44.2	173	8.7	46.8	51	2.0	*35.3*	61	6.6	50.8	134	9.0	42.5
Helmet/No	1156	8.4	49.7	684	8.9	49.7	472	7.6	*49.6*	210	10.5	60.0	719	8.6	49.1
Helmet/NA	159	5.0	37.1	109	6.4	45.0	50	2.0	*20.0*	25	4.0	40.0	110	5.5	37.3
Gender/M	1306	8.0	46.9	813	8.6	47.8	493	6.9	45.2	250	9.6	54.4	816	8.3	46.4
Gender/F	220	7.7	54.5	149	8.7	54.4	71	5.6	54.9	44	6.8	70.5	136	8.8	52.9
Age/≤ 18	450	**4.5**	**47.9**	254	**4.7**	47.1	193	4.7	49.7	73	*4.1*	57.5	286	**5.2**	47.9
Age/19–64	977	**9.7**	**49.6**	655	**10.2**	50.1	317	8.5	48.6	195	*12.3*	59.0	604	**9.9**	47.2
Age/≥ 65	39	**12.8**	**53.8**	24	**12.5**	54.2	15	13.3	53.3	11	NA	63.6	25	**20.0**	44.0
Ethnicity/Non-Hisp	666	**10.2**	**49.1**	453	**11.5**	50.0	213	7.5	46.5	113	13.3	64.6	408	**11.3**	49.0
Ethnicity/Hispanic	827	**6.2**	**47.4**	490	**5.9**	46.9	337	6.5	48.1	174	7.0	52.3	526	**6.5**	46.8

**Table 4:** Logistic regression model results for KA and KAB crashes.

		All Bicyclists (KA)			All Bicyclists (KAB)		
Variable	Reference	Estimates	Std Error	OR	Estimates	Std Error	OR
Intercept 1		−1.90 ***	0.53		−0.04	0.30	
Daylight	Dark	−0.51 *	0.22	0.6	−0.25 ^.^	0.13	0.8
Rain	No-Rain	−0.56	0.74	0.6	−0.52	0.33	0.6
Other roads	Highway/FM	−0.55 ^.^	0.29	0.6	−0.16	0.19	0.9
Weekend	Weekday	0.45 *	0.22	1.6	0.06	0.13	1.1
Speed limit >25	Speed limit ≤25	0.29	0.34	1.3	0.36 *	0.17	1.4
Turning	Straight	−0.30	0.24	0.7	0.06	0.12	1.1
8 p.m.–6 a.m.	6 a.m.–8 p.m.	0.23	0.25	1.3	−0.10	0.13	0.9
Spring	Fall	−0.04	0.29	1.0	−0.07	0.15	0.9
Summer	Fall	0.18	0.27	1.2	0.15	0.16	1.2
Winter	Fall	−0.22	0.31	0.8	−0.19	0.16	0.8
Intersection_Yes	Intersection_No	−0.39 ^·^	0.21	0.7	0.03	0.11	1.0
Intercept 2		−3.27 ***	0.35		0.19	0.17	
Helmet_Yes	Helmet_No	−0.43	0.29	0.7	−0.30 *	0.15	0.7
Helmet_NA	Helmet_No	−0.54	0.41	0.6	−0.18	0.19	0.8
Male	Female	0.10	0.28	1.1	−0.29 ^·^	0.15	0.8
Age 19–64	Age ≤18	0.78 **	0.25	2.2	0.07	0.12	1.1
Age ≥65	Age ≤18	1.16 *	0.54	3.2	0.32	0.34	1.4
Non-Hispanic	Hispanic	0.52 **	0.20	1.7	0.08	0.11	1.1

Note: *** *p* < 0.001; ** *p* < 0.01; * *p* < 0.05; “·”*p* < 0.1.

**Table 5:** Logistic regression model results for severe injury (KA) based on party at fault and bicycle facility presence.

		Bicyclist at Fault			Bicyclist Not at Fault			On Facility			Off Facility		
Variable	Reference	Estimates	Std Error	OR	Estimates	Std Error	OR	Estimates	Std Error	OR	Estimates	Std Error	OR
Intercept 1		−2.5 **	0.93		−1.71 *	0.68		−3.46 *	1.53		−2.21 **	0.74	
Daylight	Dark	−0.34	0.36	0.7	−0.57 *	0.28	0.6	−0.35	0.56	0.7	−0.59 *	0.27	0.6
Rain	No-Rain	−0.51	1.08	0.6	−0.47	1.04	0.6				−0.86	1.04	0.4
Other roads	Highway	−0.57	0.50	0.6	−0.54	0.38	0.6	0.27	1.09	1.3	−0.56	0.38	0.6
Weekend	Weekday	0.78 *	0.36	2.2	0.26	0.30	1.3	0.49	0.60	1.6	0.49 ^.^	0.28	1.6
Speed limit >25	Speed limit ≤25	0.58	0.56	1.8	0.07	0.43	1.1	−0.20	0.69	0.8	0.89 ^.^	0.54	2.4
Turning	Straight	−0.40	0.41	0.7	−0.23	0.31	0.8	−1.01	0.79	0.4	−0.08	0.29	0.9
8 p.m.–6 a.m.	6 a.m.–8 p.m.	0.58	0.47	1.8	0.17	0.32	1.2	0.85	0.68	2.3	0.07	0.31	1.1
Spring	Fall	−0.28	0.51	0.8	0.05	0.37	1.1	0.44	0.81	1.6	0.03	0.36	1.0
Summer	Fall	0.51	0.43	1.7	−0.04	0.36	1.0	0.87	0.76	2.4	0.07	0.35	1.1
Winter	Fall	0.14	0.48	1.2	−0.41	0.42	0.7	0.29	0.88	1.3	−0.3	0.39	0.7
Intersection_Yes	Intersection_No	−0.72 *	0.36	0.5	−0.15	0.27	0.9	−0.19	0.57	0.8	−0.59 *	0.27	0.6
Intercept 2		−3.26 ***	0.62		−3.32 ***	0.44		−3.88 ***	0.86		−3.04 ***	0.41	
Helmet_Yes	Helmet_No	−1.61	1.03	0.2	−0.29	0.31	0.8	−0.84	0.58	0.4	−0.24	0.34	0.8
Helmet_NA	Helmet_No	−0.97	1.04	0.4	−0.46	0.45	0.6	−0.77	1.06	0.5	−0.39	0.45	0.7
Male	Female	0.30	0.55	1.4	0.03	0.32	1.0	0.71	0.65	2.0	−0.05	0.33	1.0
Age 19–64	Age ≤18	0.71 ^·^	0.40	2.0	0.81 *	0.33	2.3	1.25 ^·^	0.86	3.5	0.66 *	0.30	1.9
Age ≥65	Age ≤18	1.29	0.85	3.6	1.09	0.70	3.0				1.52 **	0.58	4.6
Non-Hispanic	Hispanic	0.15	0.35	1.2	0.69 **	0.25	2.0	0.56	0.42	1.7	0.54 *	0.24	1.7

Note: *** *p* < 0.001; ** *p* < 0.01; * *p* < 0.05; “·”*p* < 0.1.

**Table 6:** Logistic regression model results for any injury (KAB) based on party at fault and bicycle facility presence.

		Bicyclist at Fault			Bicyclist not at Fault			On Facility			Off Facility		
Variable	Reference	Estimates	Std Error	OR	Estimates	Std Error	OR	Estimates	Std Error	OR	Estimates	Std Error	OR
Intercept 1		0.12	0.50		−0.24	0.38		−1.07	0.72		−0.10	0.38	
Daylight	Dark	−0.34	0.21	0.7	−0.20	0.16	1.0	−0.22	0.30	0.8	−0.20	0.16	0.8
Rain	No-Rain	−0.24	0.48	0.8	−0.77	0.47	0.5	−0.56	0.75	0.6	−0.95	0.45	0.4
Other roads	Highway	−0.26	0.33	0.8	−0.04	0.23	1.0	0.25	0.50	1.3	0.01	0.24	1.0
Weekend	Weekday	0.20	0.22	1.2	0.02	0.17	1.0	0.72 *	0.32	2.1	−0.06	0.17	1.0
Speed limit >25	Speed limit ≤25	0.23	0.28	1.3	0.45 *	0.22	1.6	0.01	0.38	1.0	0.47	0.22	1.6
Turning	Straight	−0.14	0.21	0.9	0.18	0.15	1.2	0.25	0.30	1.3	0.04	0.15	1.1
8 p.m.–6 a.m.	6 a.m.–8 p.m.	0.02	0.22	1.0	−0.14	0.16	0.9	0.32	0.29	1.4	−0.35	0.16	0.7
Spring	Fall	−0.12	0.25	0.9	−0.06	0.20	1.0	0.24	0.37	1.3	−0.20	0.20	0.8
Summer	Fall	0.35	0.26	1.4	0.08	0.20	1.1	0.37	0.37	1.5	0.26	0.20	1.3
Winter	Fall	−0.29	0.26	0.8	−0.12	0.21	0.9	0.07	0.40	1.1	−0.11	0.20	0.9
Intersection_Yes	Intersection_No	0.04	0.19	1.0	0.05	0.14	1.1	0.41	0.28	1.5	−0.01	0.15	1.0
Intercept 2		0.38	0.28		0.07	0.21		0.79 ^·^	0.42		0.14	0.21	
Helmet_Yes	Helmet_No	−0.61 ^·^	0.32	0.5	−0.22	0.18	0.8	−0.42	0.31	0.7	−0.36 ^·^	0.20	0.7
Helmet_NA	Helmet_No	−0.68 ^·^	0.40	0.5	−0.02	0.22	1.0	−0.32	0.49	0.7	−0.15	0.23	0.9
Male	Female	−0.30	0.26	0.7	−0.27	0.18	0.8	−0.58	0.37	0.6	−0.26	0.19	0.8
Age 19–64	Age ≤ 18	0.01	0.19	1.0	0.12	0.15	1.1	−0.07	0.29	0.9	0.08	0.15	1.1
Age ≥ 65	Age ≤ 18	0.29	0.55	1.3	0.35	0.43	1.4	0.23	0.68	1.3	−0.05	0.42	1.0
Non-Hispanic	Hispanic	−0.09	0.18	0.9	0.16	0.13	1.2	0.57 *	0.26	1.8	0.09	0.14	1.1

Note: * *p* < 0.05; “·” *p* < 0.1.

## Data Availability

Publicly available datasets were analyzed in this study. The data can be found at https://cris.txdot.gov/ (accessed on 20 March 2020).
